# NAVIGATING THE HOME CARE LANDSCAPE FOR MEDICARE BENEFICIARIES: FINDINGS FROM A SECRET SHOPPER STUDY

**DOI:** 10.1093/geroni/igae098.4029

**Published:** 2024-12-31

**Authors:** Abigail Shilvock, Peggy Leung, Madeline Sterling

**Affiliations:** Cornell University Medical College, New York, New York, United States; Weill Cornell Medicine, New York, New York, United States; Weill Cornell Medicine, New York, New York, United States

## Abstract

An estimated 2.9 million patients access home health care annually through Medicare, primarily provided by certified home health care agencies (CHHAs). Despite Medicare covering 60-day episodes of home health care, patients often experience difficulty obtaining adequate and timely care. We investigated the real-world experience of Medicare beneficiaries and their caregivers seeking care through CHHAs by conducting a cross-sectional Secret Shopper study of CHHAs in New York State between February and March 2024. A trained investigator, posing as a family caregiver seeking home care for an older adult, contacted CHHAs using a standardized script informed by the Levesque conceptual framework of access to healthcare. Out of 110 identified CHHAs, 86 were eligible for analysis. 72% of CHHAs reported availability to provide skilled home health nursing services, while 66% reported availability to provide unskilled services (i.e., home health aide). Only 5% offered private pay skilled services with nursing visits costing $115-$300 and physical therapy $145-$230 per visit. One in five CHHAs provided an hourly rate for aide services, with a mean rate of $35.60 per hour. Nearly 80% of CHHAs could obtain insurance approval and over 75% could perform an in-home nursing evaluation within a week of referral placement. However, only 50% reported the patient would start receiving services within a week of evaluation. Qualitative analysis of field notes collected by the caller revealed three main themes: 1) numerous barriers to accessing home health care, 2) variability in access and timeliness of services across agencies, and 3) inconsistent customer service.

